# Effects of red macroalgae *Asparagopsis taxiformis* supplementation on the shelf life of fresh whole muscle beef

**DOI:** 10.1093/tas/txab056

**Published:** 2021-03-19

**Authors:** Bakytzhan Bolkenov, Toni Duarte, Linghuan Yang, Frederick Yang, Breanna Roque, Ermias Kebreab, Xiang Yang

**Affiliations:** Department of Animal Sciences, University of California, Davis, Davis, CA 95616-5270

**Keywords:** *Asparagopsis taxiformis*, macroalgae, shelf life, whole muscle beef

## Abstract

This study was conducted to evaluate the effect of red macroalgae *Asparagopsis taxiformis* supplementation for cattle on the shelf life of fresh beef steaks (*longissimus dorsi*). Three treatment groups (seven steers per treatment) included: 1) Control diet, 2) Control diet + 0.25% of macroalgae inclusion (low dose, LD), and 3) Control + 0.5% of macroalgae inclusion (high dose, HD). After the animals were harvested, the strip loins from all animals were collected and aged for 14 days at the meat lab of the University of California, Davis. Then the strip loins were cut into steaks, packaged, and placed on a retail display case for 6 days. During a retail display, instrumental color (L*, a*, and b*) of lean muscle and external fat surfaces were measured every 12 h. Bacterial counts for total aerobic mesophilic bacteria (AMB), aerobic psychrotrophic bacteria (APB), and lactic acid bacteria (LAB) were assessed on days 0, 3, and 6 of retail display. The thiobarbituric acid reactive substances (TBARS) analysis was conducted to measure the lipid oxidation and the pH was also assessed on days 0, 3, and 6. No interactive effect between treatments and time on the shelf life of steaks was observed. The HD red macroalgae supplement decreased (*P* < 0.05) the lightness (L*) of the surface muscle of the steaks, while the lightness of the external fat was not affected (*P* < 0.05) by treatments throughout the retail display. The external fat yellowness of the steaks was lower (*P* < 0.05) in LD and HD treatment groups compared with the control group. An increase (*P* < 0.05) in counts of AMB, APB, and LAB was observed on the steaks from the steers in the HD treatment group while steaks in Control and LD group had similar bacterial numbers throughout the retail display. The results indicated that the shelf life of steaks from cattle in LD group remained the same as that of the Control group, but the HD of *A. taxiformis* caused a darker color of steaks with higher microbial counts, which may lead to a shortened shelf life due to undesirable appearance and faster microbial spoilage.

## INTRODUCTION

Red macroalgae are rich sources of proteins, essential amino acids, nutritious fibers, and important minerals to be used in the human diet (Galland-Irmouli et al., 1999; Mišurcová, 2012). Additionally, an evaluation of 20 species of macroalgae on in vitro fermentation showed that among the species, *Asparagopsis taxiformis* provides a promising opportunity to mitigate methane production from ruminants (Machado et al., 2014). Further studies have shown that the inclusion of red macroalgae *A. taxiformis* at different levels (0.1–5%) can reduce enteric methane production between 67% and 100% in vitro (Kinley et al., 2016; Roque et al., 2019a) and 40–98% in vivo (Li et al., 2018; Roque et al., 2019b; Kinley et al., 2020).

Meats are highly perishable products and the shelf life of meat is limited due to microbial growth, lipid oxidation, and discoloration (Lambert et al., 1991; Gray et al., 1996; Antoniewski and Barringer, 2010). The incorporation of different diet supplements can effectively extend the shelf life of meat by reducing discoloration, delaying lipid oxidation, lowering viable bacterial counts, and preventing metmyoglobin formation in meat (Ripoll et al., 2011; Guerra-Rivas et al., 2016; Chikwanha et al., 2019). A few studies have investigated the effects of different algae on meat quality and shelf life, but there is no scientific consensus among them. For instance, some studies claimed that marine algae did not change the color and lipid oxidation rate of pork (Sardi et al., 2006; Vossen et al., 2017). However, Urrutia et al. (2016) reported that marine microalgae increased lipid oxidation in lamb meat. Other studies reported that the algae supplementation improved fatty acid composition while saturated fatty acids, unsaturated fatty acids, polyunsaturated fatty acids (PUFA), and monosaturated fatty acids were not affected in beef (Hwang et al., 2014; Stokes et al., 2016). We must take into account the fact that these studies in the examples used different types of seaweeds that have different nutrient compositions.

The above-mentioned studies evaluated the effect of various types of macroalgae and microalgae on the shelf life of beef and meats of other domestic animals. However, there is no literature available evaluating the effect of *A. taxiformis* on beef shelf life and meat quality. Therefore, before incorporating *A. taxiformis* as a methane-reducing feed additive in beef cattle diets on a large scale, the effect it has on the shelf life of whole muscle beef must be evaluated. The objective of this study was to assess the change of color, microbial count, pH, and lipid oxidation in beef from cattle supplemented with different doses of *A. taxiformis*, during the retail display.

## MATERIALS AND METHODS

All animal use protocols were approved by UC Davis’ Institutional Animal Care and Use Committee (IACUC). The protocol number is 20803.

### Animals, Diet, and Experimental Design

A total of 21 Angus/Hereford steers were individually fed for 21 weeks and individually housed at the feedlot of the University of California, Davis. Animals were first blocked by weight then randomly assigned to one of three treatment groups: Control (basal diet), Low Dose (LD; basal diet + 0.25% *A. taxiformis* inclusion on an organic matter basis), and High Dose (HD; basal diet + 0.50% *A. taxiformis* inclusion on an organic matter basis). The *A. taxiformis* used in this study was mixed with 200 mL of molasses and 200 mL water then hand-mixed into the feed twice a day. The Control group received the same molasses and water mix to decrease dietary variability between treatment groups. The animals were on high forage (starter diet) for 9 weeks, on mid forage (transition diet) for 3 weeks, and low forage (finisher diet) for 9 weeks. The low and high dosages of *A. taxiformis* were incorporated into these diets and were given to the treated animal groups. The ingredients of the high, mid, and low forage diets are presented in Table 1. Nutritional composition of experimental diets, *A. taxiformis*, and alfalfa pellets are presented in Table 2.

**Table 1.** Composition of the starter, transition, and finisher diets

Ingredients (% of DM)	Starter diet	Transition diet	Finisher diet
Alfalfa hay	35.0	25.0	5.00
Wheat hay	25.0	15.0	6.00
Dry distillers grain	12.0	14.0	6.00
Rolled corn	20.0	37.0	72.0
Molasses	5.00	5.00	3.00
Fat	1.50	2.00	3.00
Urea	0.35	0.40	1.80
Beef trace salt	0.32	0.32	1.00
Calcium carbonate	0.82	1.15	1.80
Magnesium oxide			0.20
Potassium chloride			0.50

**Table 2.** Nutritional composition of experimental diets, *A. taxiformis*, and alfalfa pellets

	High forage	Medium forage	Low forage	Pellets	*A. taxiformis*
% Dry matter					
Organic matter	92.1	93.1	94.8	88.6	50.9
Crude protein	17.2	17.4	13.2	17.1	16.8
ADF	22.6	16.7	10.5	28.1	11.5
NDF	33.1	25.8	18.6	35.9	33.7
Lignin	4.08	3.05	1.73	6.16	4.08
Starch	16.9	25.0	46.7	0.90	0.35
Crude fat	4.92	6.04	6.77	3.02	0.63
Calcium	0.77	1.00	0.50	2.06	5.29
Phosphorus	0.33	0.38	0.28	0.24	0.18
Magnesium	0.38	0.38	0.23	0.37	0.81
Potassium	1.74	1.42	0.94	2.10	2.02
Sodium	0.18	0.25	0.30	0.20	6.34
Parts per million					
Iron	438	335	127	1,508	8,494
Manganese	61.7	56.0	64.0	88.0	142.5
Zinc	43.2	51.50	58.0	19.0	53.5
Copper	8.67	8.00	7.00	10.0	22.5

The seaweed was collected by the Center for Macroalgal Resources and Biotechnology of James Cook University, Townsville, Queensland, Australia. Once collected, the *A. taxiformis* biomass was frozen and stored at −15°C then freeze–dried and ground (2–3 mm).

### Sample Preparation

After the completion of the feeding trial, cattle were harvested at a commercial packing plant in northern California. Strip loins (Institutional Meat Purchase Specification 180; *longissimus dorsi*, *n* = 21) were collected from the left side of the carcasses (USDA Prime and Choice) and vacuum-sealed in vacuum bags (Cryovac Barrier Bags, Model B6620, Sealed Air Corporation, Charlotte, NC); MVTR: 0.35–0.45 g/(100 in.^2^/24 h/1 atm @38°C; 100%RH) and OTR 2.0–4.5 cc/(m^2^/24 h/1 atm @ 5°C, 0%RH) under refrigerated conditions. Further, strip loins were wet aged at 4–6°C in dark for 14 days. After aging, each strip loin was cut from anterior to posterior into several (12–13 steaks) 2.54 cm steaks. Three randomly selected steaks from each strip loin were individually put on drip pads (Classic pad; Tite-Dri Industries, Boynton Beach, FL), packed on a foam tray (2 W Foam Tray; CKF Inc., Toronto, Canada) and overwrapped using polyvinyl chloride (PVC) film (Berry AEP 1504311 18″ Perforated 40 Gauge PVC Film Shrink Wrap; AEP Industries Inc., Montvale, NJ). The water vapor transmission rate of the PVC film is 24 g/100 sq. in./24 h while oxygen transmission rate is 825 cc/100 sq. in. in 24 h. The steaks were placed in a retail display case (C2NX4XLEPM Multi-Deck; Hussmann Corp., Bridgeton, MO) at a temperature of 4°C and exposed to light (LED-florescent light bulb, 3500 K) all the time for 6 days. One steak from the three steaks was chosen randomly for day 6 sample and used for the color measurements. Steak samples were shuffled on the shelves every 12 h to reduce the variance caused by light and temperature. The light intensity in the display case was measured every 12 h using a light meter (Heavy Duty Datalogging Light Meter, model HD 450; Extech instruments, Nashua, NH). The measured light intensity at the display ranged from 512 to 763 lux with an average of 688 lux. The temperature of the retail case was monitored using temperature data loggers (Console Pro; Cryopak, Monticello, AR).

### Instrumental Color Measurement

Instrumental color was measured at three positions on the surface lean and external fat of each steak using an illuminant A/10° observer with a portable spectrophotometer (Hunter MiniScan XE, model 45/O-S; Hunter Associates Laboratory Inc., Reston, VA) every 12 h daily for 6 days. The CIE L* (lightness), a* (redness), and b* (yellowness) values were averaged and recorded for statistical analysis. The spectrophotometer was calibrated using the black glass and white tile before each sampling point. The formula [arctangent(b*/a*)] was used to calculate hue angle (true redness) and chroma (color saturation) was calculated using [sqrt(a* ^2^ + b* ^2^)] formula (Hunt et al., 1991).

### Microbial Analysis

Bacterial counts for total aerobic mesophilic bacteria (AMB), aerobic psychrotrophic bacteria (APB), and lactic acid bacteria (LAB) were assessed on days 0, 3, and 6 of retail display. On each sampling day, about 50 g of lean meat from each steak was aseptically excised, cut into 1 cm × 1 cm cubes, and placed into a Whirl-Pak filter bag (Nasco, Modesto, CA). Buffered peptone water (BPW; 0.1%; Difco; Becton, Dickinson and Company, Sparks, MD) with a 1:2 ratio (w:w) was added to the sample and homogenized using a paddle lab blender (Masticator Silver Panoramic, Neutec Group Inc, Farmingdale, NY) for 2 min. The homogenized samples were serially diluted and spread plated in duplicate on tryptic soy agar (Difco) for AMB and APB, and pour plated on Lactobacilli MRS Agar (Difco) for LAB. The plates for AMB and LAB were incubated at 25°C for 48 h and APB plates were incubated at 7°C for 10 days, respectively. After the incubation period, all the plates were counted. The results were reported as log CFU/g.

### pH Measurement

On each sampling day, 5–10 g of muscle from each steak was excised and mixed with distilled water (5× of muscle weight) in a non-filtered Whirl Pak bag (Nasco, Modesto, CA) using a paddle blender-masticator (Neu-tec Group Inc; Lenox Avenue Farmingdale, NY). The pH of each sample was measured using a benchtop pH meter (Oakton pH 700 Benchtop Meter; Cole-Parmer, Vernon Hills, IL) at room temperature. The pH meter was calibrated with standard solutions (pH = 7.0 and 4.0) before measuring the samples.

### TBARS Analysis and Fat Composition

The remaining lean meat (the combination of surface and interior) from each steak used for microbial analysis on days 0, 3, and 6 was cut into small pieces and stored at −80°C for subsequent TBARS test within 1 month. On the day of analysis, stored samples were taken out from the freezer, put into liquid nitrogen, and pulverized using a blender (Magic bullet; Capbran holdings LLC, Los Angeles, CA). The TBARS analysis was performed following the procedure described by Buege and Aust (1978). Lipid oxidation was determined by quantifying the malonaldehyde (MDA) concentration in each sample. The results of TBARs were expressed as mg MDA per kg of meat.

Frozen beef steaks from all the animals were sent for crude fat and fatty acid analyses (Midwest Labs, Omaha, NE). The crude fat content was fat measured following AOAC Official Method 991.36 (2012). The fatty acids profile was determined according to the protocol of AOAC Official method 996.06 (2001).

### Statistical Analysis

The experiment for shelf life study was a split-plot design. For pH, microbial, and TBAR analyses, the data were analyzed using two-way analysis of variance (ANOVA) to investigate the diet treatment effect (Control, LD, HD), the day effect (days 0, 3, 6), and their corresponding interactions. The color data were analyzed using one-way ANOVA with repeated measures to evaluate the effect of diet treatment (Control, LD, HD), time (every 12 h from day 0 to day 6), and their corresponding interaction. The fat content and fatty acids data were analyzed using one-way ANOVA to test the main effect of the diet treatment. Data were analyzed using packages ANOVA, Emmeans, and Cld in R statistical software (version 3.6.1; The R Foundation for Statistical Computing, Vienna, Austria). Statistical significance was tested at an alpha level of 0.05.

## RESULTS AND DISCUSSION

### Objective Color

No interaction between treatments and display time for objective color was observed. Steaks from HD treatment showed lower (*P* < 0.05) L* values of the lean surface compared with LD treatment and control throughout the retail display, indicating that steaks from HD treatment had a darker appearance during the retail display. On day 0, the lean L* values for the steaks across all three treatment groups were higher than the values obtained from the following days while on day 6 of the retail display, the L* values were reduced (*P* < 0.05) for the steaks from all three treatment groups. Both HD and LD treatments did not affect (*P* < 0.05) a* and b* values for lean muscle. Hue angle and chroma values are presented in Table 3. At day 6, hue angle values were higher (*P* < 0.05) for all the treatment groups compared with initial day 0 values, indicating that the steaks were less red on the last day of retail display. Chroma values were higher (*P* < 0.05) on day 0 compared with the last day (day 6), indicating that color became dull as retail display days increased.

**Table 3.** Least square means for traits of steaks from cattle with different diet treatments stored in the retail display case at 4°C

Traits	Control	LD*	HD^*^	SEM	*P*-value
Lean L* ^†^	44.7^a^	44.3^a^	43.3^b^	0.23	<0.05
Lean a* ^†^	30.6	30.7	30.9	0.31	0.72
Lean b* ^†^	26.3	26.3	26.6	0.27	0.71
Hue angle	40.8	40.8	40.7	0.21	0.93
Chroma	40.4	40.5	40.8	0.38	0.69
Fat L*	73.3	73.1	71.2	0.67	0.07
Fat a*	11.5^a^	9.5^b^	10.4^ab^	0.54	0.03
Fat b*	17.5^a^	15.6^c^	16.7^b^	0.24	<0.05
AMB, log CFU/g^‡^	6.16^b^	6.58^ab^	6.79^a^	0.15	0.05
APB, log CFU/g^‡^	6.18^b^	6.55^ab^	6.79^a^	0.15	0.03
LAB, log CFU/g^‡^	5.78^b^	5.99^ab^	6.29^a^	0.14	0.05
pH	5.46	5.47	5.48	0.01	0.64
TBARS, mg MDA/kg^||^	0.57	0.59	0.56	0.50	0.78

^*^Treatment containing LD and HD of *A. taxiformis* supplementation.

^†^Lightness(L*), redness (a*), and yellowness (b*) of beef muscle and external fat color of the steaks.

^‡^AMB, APB, and LAB.

^||^TBARS, thiobarbituric acid reactive substances analysis.

^a,b,c^Least square means (seven samples/treatment for beef steaks) within a row with different superscripts differ (*P* < 0.05). There was no interactive effect between treatments and storage time, so the means are averaged by storage time.

The lightness of the external fat of the steaks was not (*P* < 0.05) affected by the inclusion of *A. taxiformis*. Animals from both LD and HD treatments produced meat with less yellow fat (*P* < 0.05) when compared with those from the Control group. Also, the b* values of the LD treatment group were lower than the HD treatment group. The a* values for the external fat were lower (*P* < 0.05) for the steaks from the LD treatment group when compared with steaks of the HD and Control groups (Table 3).

Our results indicated that the steaks from the HD treatment group had a darker lean surface during the 6 days of retail display than those of the LD and Control treatment groups. The results were supported by other studies. For example, Phelps et al. (2016) detected that the ground meat patties made from the knuckle cuts of heifers fed with microalgae meal *Schizochytrium limacinum* at different inclusion level (0, 50, 100, and 150 g·heifer^−1^·d^−1^ of microalgae meal) had lower L* values on day 0 than the Control group. Additionally, Michalak et al. (2020) observed a slight darkening in meat (*longissimus dorsi*) from the pigs fed macroalga *Enteromorpha* spp. In contrast, Rajauria et al. (2016) reported that fresh chops (*longissimus dorsi*) from pigs treated with the seaweed extract showed a brighter appearance than the control group during a 14-day storage period when the steaks were stored in the modified atmosphere packs with 80% O_2_:20% CO_2_. The authors concluded that the high antioxidant radical scavenging capacity of the seaweeds protected the animals from oxidative stress which in turn helped to stabilize the redness of meat samples after the slaughter.

In the present study, the a* values of the steaks between the treatment groups were similar, suggesting that the redness of the beef steaks were not affected by the incorporation of *A. taxiformis* in cattle diet. This finding agreed with Moroney et al. (2012) where the redness of fresh minced pork (*Musculus longissimus dorsi*) was not affected by a dried seaweed (*Laminaria digitata*) extract in pig feed at concentration levels of 0.01%, 0.1%, and 0.5%. Also, the addition of macroalga *Enteromorpha* sp. into the diets of pigs did not change the redness of *longissimus dorsi* (Michalak et al., 2020). However, Phelps et al. (2016) reported that ground meat patties from heifers treated with the microalgae meal had lower a* values compared with the control group. Since consumers always link cherry/pink red color to the freshness of the beef/pork (McMillin, 2008), producers need to supplement the appropriate seaweed/algae in the animal diet that should not reduce the redness of the steaks, which may be rejected by the consumers.

### Microbial Counts

There was no (*P* < 0.05) interactive effect between the time and treatment on AMB, APB, and LAB count. The average initial microbial counts on day 0 for AMB, APB, and LAB were between 4.37 and 4.97 log CFU/g for Control group, between 4.88 and 5.23 log CFU/g for LD treatment group, and between 4.82 and 5.22 log CFU/g for HD treament group (Table 4). However, on day 6, the bacterial population for all types exceeded 7 log CFU/g in all the steaks across treatments. Counts for AMB, APB, and LAB were higher (*P* < 0.05) in the steaks from the HD treatment group compared with Control while there were no differences between the LD treatment steaks and control steaks during the retail display.

**Table 4.** Least squares mean of bacterial counts for beef steaks during the retail display at 4°C

	AMB, log CFU/g^*^				APB, log CFU/g^*^				LAB log CFU/g^*^			
Day	Control	LD^†^	HD^†^	Average	Control	LD^†^	HD^†^	Average	Control	LD^†^	HD^†^	Average
0	4.37	4.88	4.93	4.72^c^	4.54	5.02	4.82	4.79^c^	4.97	5.23	5.22	5.13^c^
3	6.06	6.56	6.98	6.53^b^	6.00	6.24	7.01	6.41^b^	5.35	5.70	6.19	5.75^b^
6	8.05	8.30	8.45	8.27^a^	8.00	8.39	8.23	8.20^a^	7.06	7.05	7.38	7.16^a^
SE	0.15	0.15	0.16	0.15	0.15	0.15	0.15	0.15	0.14	0.14	0.15	0.15

^*^AMB, APB, and LAB.

^†^Treatments containing LD and HD of *A. taxiformis* supplementation.

^a,b,c^Least square means (7 samples per treatment per day) within a column with different superscripts differ (*P* < 0.05).

In our study, higher counts of AMB, APB, and LAB were observed in the steaks from the HD treatment group steers. Results from the present study supported the findings of another study that the addition of dried *Himanthalia elongata* (5%) into the pork frankfurters caused an increased microbial population (López-López et al., 2009). There is no literature reporting an increase of microbial population in meat when animals were fed seaweeds. However, Rajauria et al. (2016) reported that *longissimus dorsi* steaks from the pigs fed with seaweed extract exhibited lower total viable counts until day 10 than the control group under refrigerated conditions. The researchers concluded that the prebiotic properties of the supplement are the cause of the difference in bacterial load. In the same study, the steaks were stored in modified atmosphere packages (200 × 300 mm, McDonnells Ltd., Dublin, Ireland) with 80% O_2_:20% CO_2_, which has also been shown to inhibit bacterial growth (Lambert et al., 1991).

Moreover, other studies found that the extract from brown seaweed (*L. digitata*) exerted no effect on the microbial population in fresh *longissimus dorsi* muscle of porcine during the retail storage (Moroney et al., 2012, 2013). However, many studies reported the antimicrobial functions of brown seaweed polysaccharides such as laminarin and fucoidan (Costa et al., 2010; Gupta and Abu-Ghannam, 2011; Cox et al., 2014).

According to the data (Table 3), the aerobic bacteria (AMB and APB) counts were higher (*P* < 0.05) in the HD treatment group. Microbial spoilage might be the reason of faster deterioration of the HD steaks. According to Lambert et al. (1991), microorganisms are the most important factor that affects meat color. High oxygen utilization of aerobic bacteria in their growth phase causes oxygen tension on the surface of meat and bacterial discoloration occurs as the result deficit of oxygen in meat (Butler et al., 1953).

### pH

No (*P* < 0.05) difference in pH was found among treatments (Table 3). However, over the retail display time, the pH increased (*P* < 0.05) from 5.42 to 5.51 across all three treatment groups. In the present study, color differences were detected without the presence of significant differences in meat pH.

### Fat Composition and TBRAS Values

Total fats, saturated fatty acids, PUFA, and monounsaturated fatty acids (MUFA) concentrations were not different among the treatment groups (Table 5). In addition, there was no interactive effect of treatment by days, and neither a treatment effect detected for TBARS values (Table 3). At the start of the display period (day 0) TBARS values were ranged from 0.31 to 0.35 mg MDA/kg across samples. However, on day 6, the TBARS values for all samples ranged between 0.88 and 0.91 mg MDA/kg which exceed the oxidative rancidity threshold of 0.5 mg MDA/kg that was established by Tarladgis et al. (1960).

**Table 5.** Fat contents of the steaks from the steers fed Control, LD (0.25%) and HD (0.5%) inclusion levels of *A. taxiformis* diet

Fat contents	Control	LD	HD	SEM	*P*-value
Total Fat, %	8.66	7.33	6.67	0.56	0.06
Saturated fatty acid (SFA), %	48.9	50.4	49.9	0.52	0.16
Polyunsaturated fatty acid (PUFA), %	2.24	2.45	2.15	0.13	0.27
Monounsaturated fatty acid (MUFA), %	48.5	47	47.47	0.51	0.14
PUFA:SFA ratio	0.046	0.048	0.043	0.002	0.38

Lipid oxidation is one of the main factors leading to chemical deterioration of stored meat and meat products (Ladikos and Lougovois, 1990). In the present study, the degree of lipid oxidation of the steaks was not affected by the diet supplement of *A. taxiformis*. However, a study (Moroney et al., 2012) reported a slower lipid oxidation rate of fresh pork from pigs fed with the extract of brown macroalgae (*Laminaria digitate*) at an inclusion rate of 500 mg/kg feed compared with the control group. The reason behind their findings might be the fact that *L. digitata* is a good source of several bioactive compounds and a good source of antioxidant metabolites (O’Sullivan et al., 2010; Makkar et al., 2016). The inclusion of antioxidants into animals’ diet retards lipid oxidation of meat derived from these animals (Li and Liu, 2012). On the contrary, marine microalgae (DM basis; Market DHA Gold, *Schizochytrium* spp.; Market Biosciences Corp., Columbia, MD) at inclusion dose of 3.84% increased the lipid oxidation of the lamb meat (Urrutia et al., 2016). The same pattern was reported by Hopkins et al. (2014) that lambs fed with DHA-Gold algae supplement (~2% of the ration) showed a higher rate of lipid oxidation compared with the control group. The fast rate of lipid oxidation in these studies might be due to the high concentration of docosahexaenoic acid in the *Schizochytrium* spp. and in the DHA-Gold algae, since docosahexaenoic acid is PUFA which has a greater potential for lipid oxidation in meat (Hausman et al., 2009).

The findings of Stokes et al. (2016) are similar to our results that concentrations of saturated, monosaturated, and PUFA in the steaks were not affected by the macroalgae supplementation in animal diet. By contrast, the ground beef from heifers fed with microalgae meal (All-G Rich; *Schizochytrium limacinum* CCAP 4067/2) had greater concentrations of PUFA which decreased the lightness and redness of ground beef (Phelps et al., 2016). In this study, concentration of seaweeds in the diet was much higher (concentration of Schizochytrium spp. in the diet was up to 150 g/day) compared with doses of seaweed applied in our study, which suggested that a high dosage of algae in animal diet is associated with increased concentration of PUFA that ultimately accelerates lipid oxidation rate (Hopkins et al., 2014). The above-mentioned correlation between PUFA concentration and lipid oxidation rate was evident in our study, where neither PUFA concentration nor lipid oxidation rate was affected by *A. taxiformis* supplementation in cattle diet.

In conclusion, our findings indicate that the inclusion of the LD of *A. taxiformis* (0.25%) into the cattle diet did not have adverse effects on the microbial and physicochemical characteristics of beef steaks during retail display. However, the HD of *A. taxiformis* (0.5%) added to the cattle diet lowered the brightness of muscle and increased the microbial population in the steaks, which potentially shortened the shelf life of the steaks due to undesirable appearance and faster microbial spoilage.
